# Evaluation of the Quantitative Accuracy of 3D Reconstruction of Edentulous Jaw Models with Jaw Relation Based on Reference Point System Alignment

**DOI:** 10.1371/journal.pone.0117320

**Published:** 2015-02-06

**Authors:** Weiwei Li, Fusong Yuan, Peijun Lv, Yong Wang, Yuchun Sun

**Affiliations:** 1 Center of Digital Dentistry, Peking University School and Hospital of Stomatology, Beijing, China; 2 Faculty of Prosthodontics, Peking University School and Hospital of Stomatology, Beijing, China; 3 National Engineering Laboratory for Digital and Material Technology of Stomatology, Beijing, China; 4 Research Center of Engineering and Technology for Digital Dentistry, Ministry of Health, Beijing, China; Xiamen University, CHINA

## Abstract

**Objectives:**

To apply contact measurement and reference point system (RPS) alignment techniques to establish a method for 3D reconstruction of the edentulous jaw models with centric relation and to quantitatively evaluate its accuracy.

**Methods:**

Upper and lower edentulous jaw models were clinically prepared, 10 pairs of resin cylinders with same size were adhered to axial surfaces of upper and lower models. The occlusal bases and the upper and lower jaw models were installed in the centric relation position. Faro Edge 1.8m was used to directly obtain center points of the base surface of the cylinders (contact method). Activity 880 dental scanner was used to obtain 3D data of the cylinders and the center points were fitted (fitting method). 3 pairs of center points were used to align the virtual model to centric relation. An observation coordinate system was interactively established. The straight-line distances in the X (horizontal left/right), Y (horizontal anterior/posterior), and Z (vertical) between the remaining 7 pairs of center points derived from contact method and fitting method were measured respectively and analyzed using a paired *t*-test.

**Results:**

The differences of the straight-line distances of the remaining 7 pairs of center points between the two methods were X: 0.074 ± 0.107 mm, Y: 0.168 ± 0.176 mm, and Z: −0.003± 0.155 mm. The results of paired *t*-test were X and Z: *p* >0.05, Y: *p* <0.05.

**Conclusion:**

By using contact measurement and the reference point system alignment technique, highly accurate reconstruction of the vertical distance and centric relation of a digital edentulous jaw model can be achieved, which meets the design and manufacturing requirements of the complete dentures. The error of horizontal anterior/posterior jaw relation was relatively large.

## Introduction

When performing complete denture and dental occlusal rehabilitation therapy in edentulous patients, the use of an edentulous gypsum model and occlusal bases to accurately reproduce physiological jaw relation, such as vertical distance and horizontal relation, is the main basis for the design and manufacturing of suitable and personalized artificial dentition.

When using computer-aided design (CAD) and manufacturing techniques for denture production, a computerized three-dimensional (3D) virtual reconstruction of jaw relation in upper and lower edentulous jaw models and occlusal bases is required. The reconstruction error directly determines CAD accuracy for complete denture. Previous studies have indicated that the use of a model spatial relation positioning device supported by a dental model 3D scanner (dental articular positioning) and the common regional registration method can control the reconstruction error of jaw relation within one hundred microns [1, 2]. However, this method requires respective completion of five scanning operations on the upper jaw model, upper jaw dentures, lower jaw model, lower jaw dentures, and double jaw model/dentures of the complete labial and buccal surface. In addition, models need to be sequentially installed in a mechanical dental articulator for subsequent positioning, therefore the operation procedures are complicated and error prone with less efficiency.

## Materials and Methods

### 1 Experimental equipment and software

Materials used were as follows: red and white proofing cream (Shanghai Pinnacle Dental Materials Co., Shanghai, China), dental alginate impression materials (Heraeus Kulzer Dental Ltd., Shanghai, China), Heraeus Kulzer Die-Stone (Heraeus Kulzer, LLC, South Bend, IN, USA), oral opacifier (Yeti Dental, Engen, Germany).

Equipment used were as follows: computer with Intel Core i5-3550 Processor (Intel, Santa Clara, CA, USA) with 8GB memory, 1TB hard disk drive, and color display (VG920, ViewSonic, Walnut, CA, USA), 3D resin printer (EnvisionTEC, Gladbeck, Germany) with a layer accuracy of 0.025 mm, jaw model 3D scanner (Activity 880; SmartOptics, Bochum, Germany) with an accuracy of 0.010 mm, mechanical 3D measurement arm (Faro Edge 7-axis contact measurement system; Faro, Lake Mary, FL, USA) with contact measurement accuracy of 0.024 mm.

Software used were as follows: jaw model 3D scanner supporting software (Activity v2.6; SmartOptics, Germany), NX Imageware 13 software (Siemens, Germany), CAM2 Measure 10 software (Faro, USA), Geomagic Studio 2012 software (Raindrop Geomagic, USA), and SPSS 13.0 statistical software (IBM, USA).

### 2 Experimental methods

**2.1 Ethics Statement**. The study was approved by the Bioethics Committee of Stomatological Hospital of Peking University, Beijing, China. (No.PKUSSIRB-2013010. Date: 28/1/2013). We obtained written informed consents from all the participants in our study.

**2.2 Model preparation**. One edentulous patient admitted to the Department of Prosthodontics at Peking University School and Hospital of Stomatology, Beijing, China was enrolled and gave informed consent. In accordance with the requirements of a prosthodontics textbook [3], maxillary and mandibular impressions of the patient were prepared following a two-step impression method, and a gypsum model was then poured into a standard model base. A set of occlusal bases was then prepared.

**2.3 Positioning cylinder preparation and application**. Positioning cylinders of the same size (base plane diameter of 6 mm, height of 5 mm) were designed and prepared using a 3D resin printer. Ten positioning cylinders were adhered to the base axial surface of each jaw model; therefore, each model had 20 positional cylinders that were arranged as evenly as possible.

According to the dental notation from the Fédération Dentaire Internationale (FDI), the 20 positional cylinders of each model were numbered as 11–15, 21–25, 31–35, and 41–45. The center points at the base plane of positional cylinders were paired, and 10 center point pairs were defined: 11–41, 12–42, 13–43, 14–44, 15–45, 21–31, 22–32, 23–33, 24–34, and 25–35. The distances between paired points in the horizontal left/right, anterior/posterior, and vertical directions were used as quantitative indicators for evaluating the spatial relation between the upper and lower jaw models (jaw relation).

**Fig 1:**
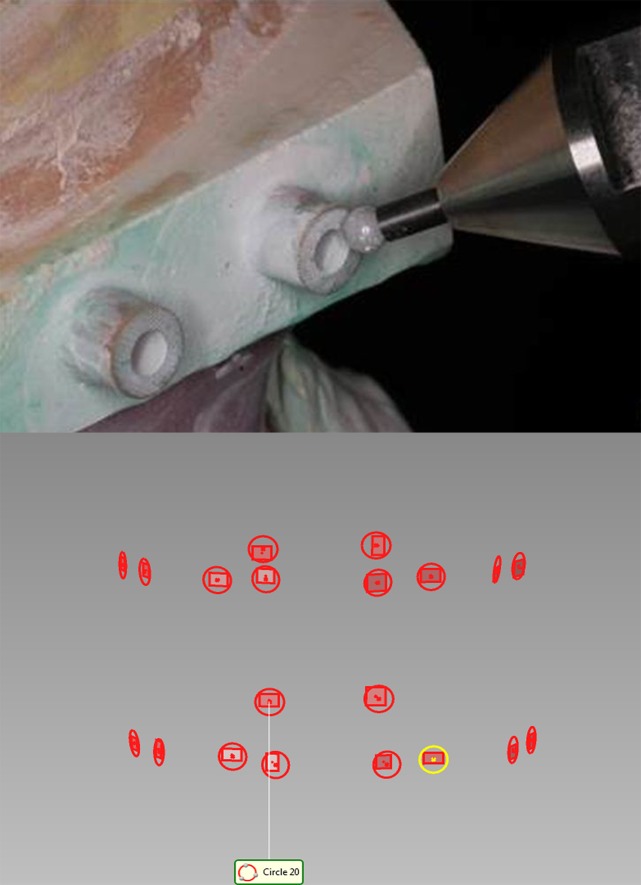
Determination of contact method center points.

**2.4 3D reconstruction of the digital model of edentulous jaw relation**. The occlusal bases and upper and lower jaw models were fixed in the centric relation position. The base and side surface of the 20 positioning cylinders of the upper and lower jaw gypsum models were directly measured using 3D contact measurement system, and respectively fitted as flat and cylindrical surfaces. Overlapping circles at the base surface of positioning cylinders were obtained after intersecting to generate center points, named ‘contact method center points’ (Fig. 1).

**Fig 2:**
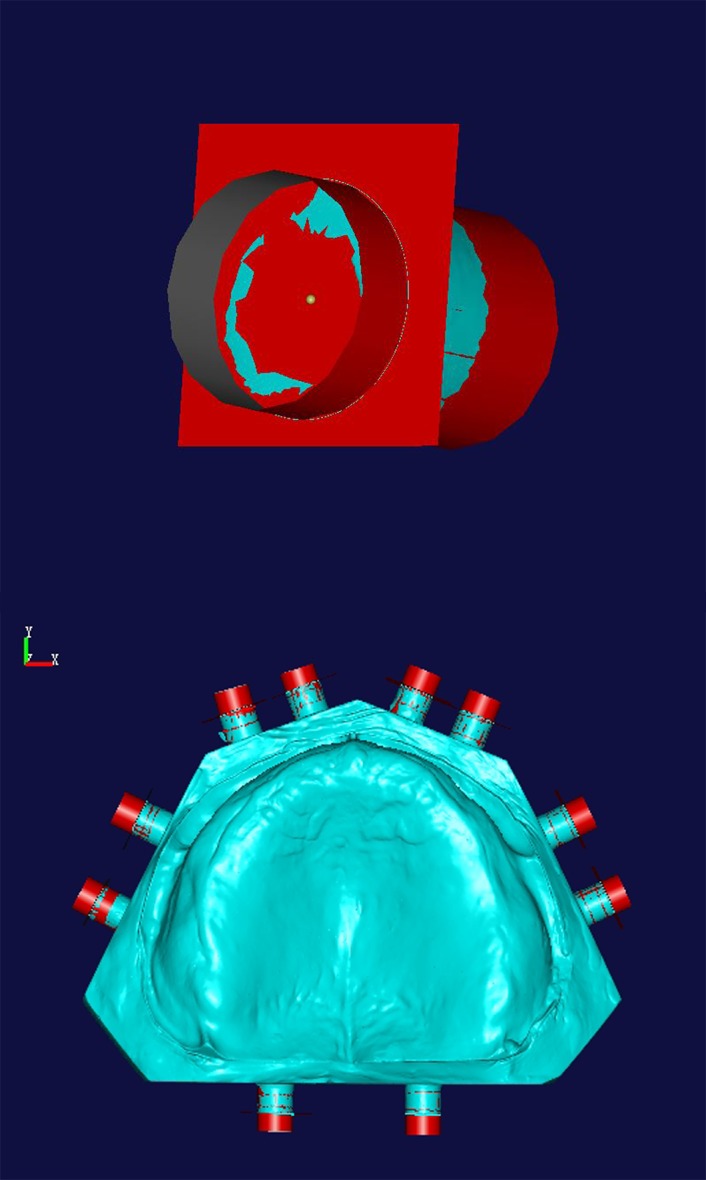
Determination of fitting method center points.

The occlusal bases were evenly sprayed with an opacifier specific for 3D scanners. Upper and lower jaw models and occlusal bases were scanned to obtain digital models A (data for the upper jaw model and occlusal base) and B (data for the lower jaw model and occlusal base). Models A and B were imported into NX Imageware 13. The base and side surface of positioning cylinders were generated by fitting, and overlapping circles at the base surface were obtained after intersecting. By using the software’s ‘Construct-point, the center point’ command, 20 center points were generated and named ‘fitting method center points’ (Fig. 2). The data were stored in STL file format.

**Fig 3:**
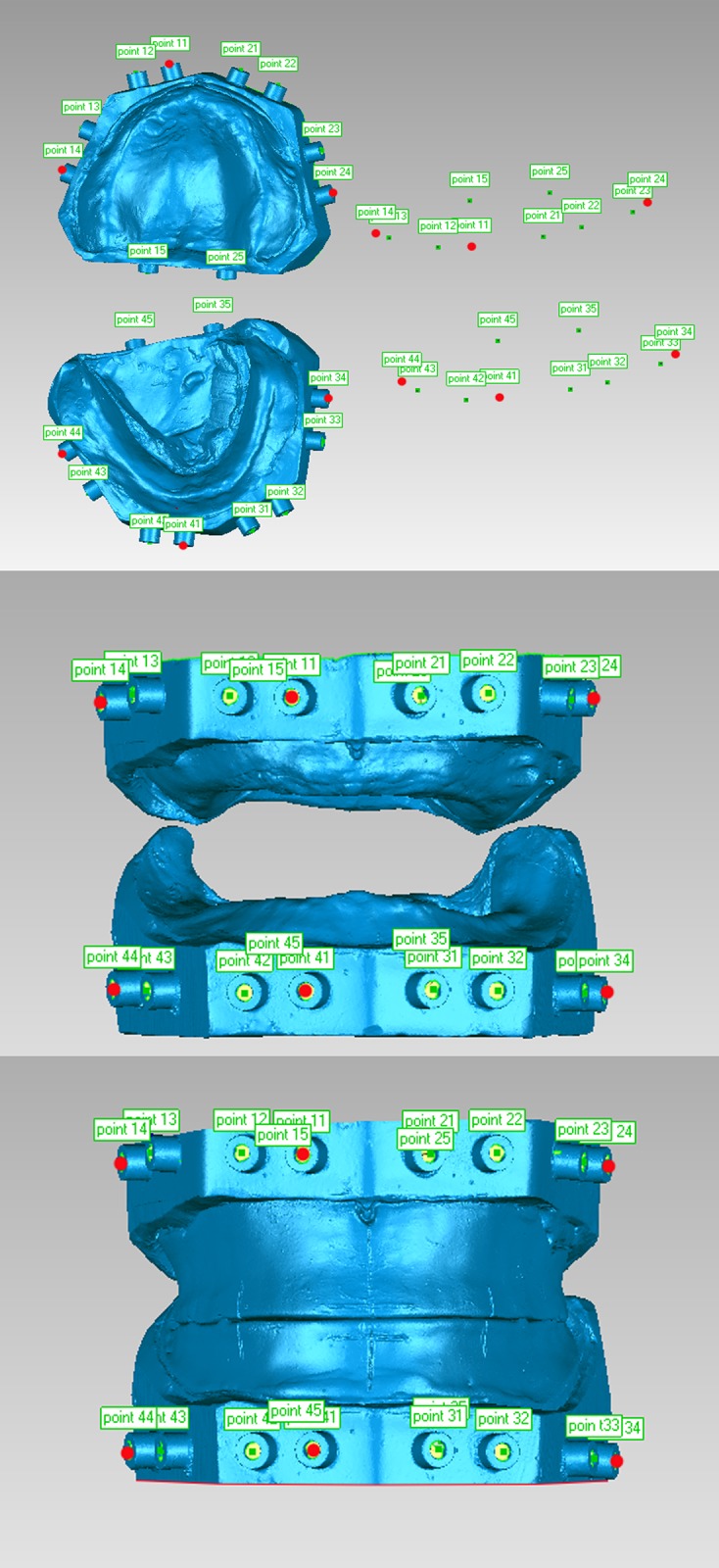
Registration of mark points for upper and lower edentulous jaw models and dentures using the RPS registration command.

By using the aforementioned two methods, center points of three positioning cylinders near the first molars and central incisor of a single jaw were obtained and considered as paired registration mark points. By using the RPS registration command in Geomagic 2012, contact method center points were respectively registered for digital models A and B. Three points, 11, 14, and 24, were registered for the upper jaw model, and three points, 41, 44, and 34, were registered for the lower jaw model. By using this method, 3D reconstruction of an edentulous jaw model and denture jaw relation was established (Fig. 3).

**Fig 4:**
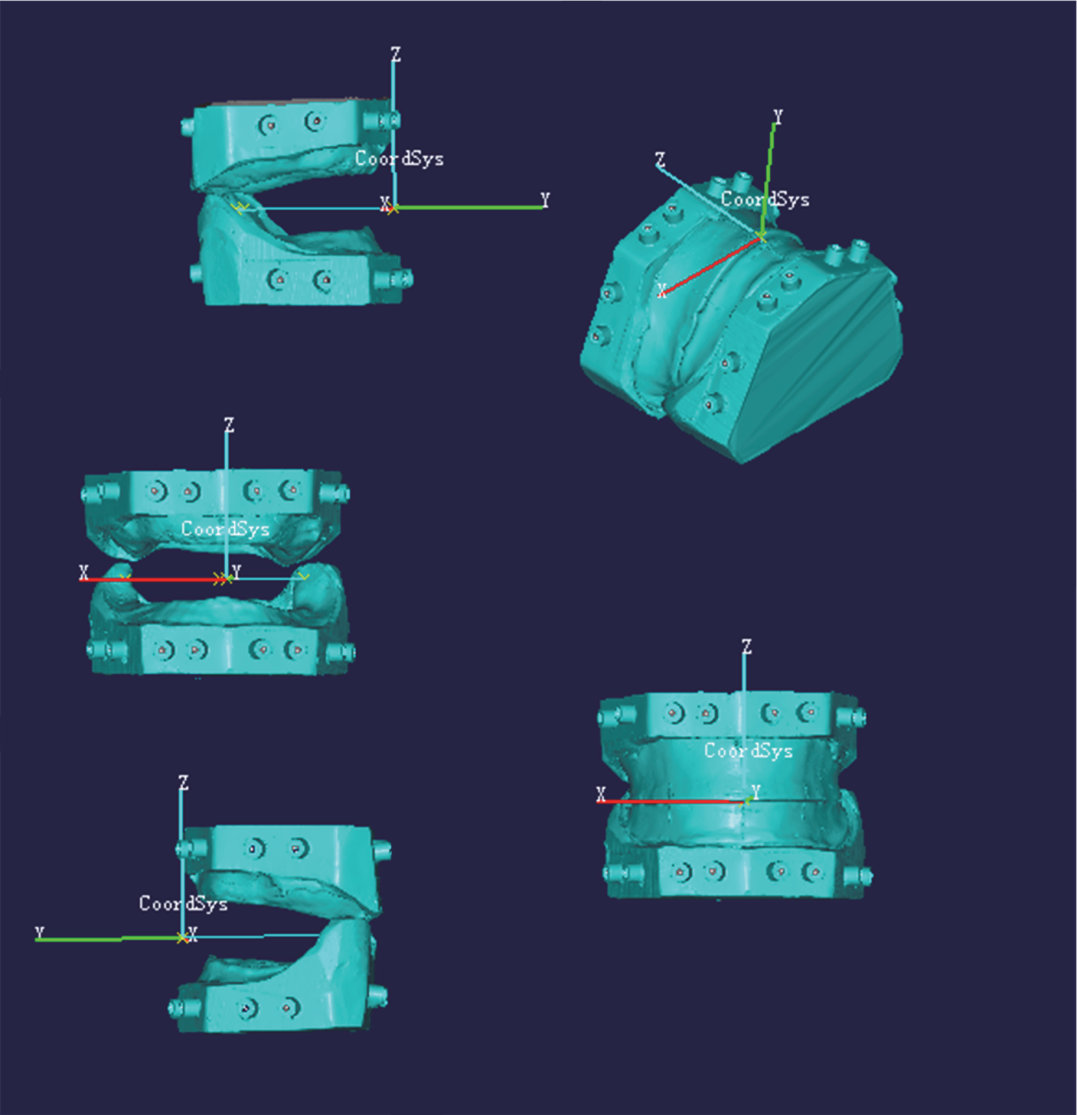
Observation coordinate system.

**2.5 Model measurement**. The registration data were imported into NX Imageware 13. The intersecting line of the labial/buccal surface and occlusal surface was selected, and the point of intersection O of this line and the midline of the upper jaw labial surface was determined. A plane was determined based on three points, point O and the bucco-lingual midpoints of the retromolar pads in the lower jaw. This was called the XOY plane, and point O was the coordinate origin. The Y-axis was the line between the midpoint between bilateral retromolar pads and point O, and an “observation coordinate system” (Fig. 4) was established based on Descartes’ rule of signs. The X-axis of the coordinate system was horizontal left/right, the Y-axis was horizontal anterior/posterior, and the Z-axis was vertical, which respectively represented horizontal left/right, anterior/posterior, and vertical jaw relations between the upper and lower edentulous jaw models.

In the coordinate system, in addition to the registration mark points, the ‘distance-measuring-point’ command was used to respectively measure the straight-line distance of the remaining seven pairs of center points in the contact and fitting methods in three directions, X, Y, and Z. The difference between these two methods was calculated as the reconstruction error of jaw relation.

**2.6 Statistical analysis**. SPSS 13.0 software was used for statistical analysis. The straight-line distances between the paired center points of these two methods in three directions, X, Y, and Z, were analyzed using a paired *t*-test. The statistical significance level was bilateral α = 0.05.

## Results

Table 1 shows the three pairs of reference center points, and the mean absolute value of the difference of the straight-line distance between the contact method and fitting method was 0.0034 mm.

**Table 1:** Measurement values and differences of the three pairs of reference center points (unit: mm).

	11–41	14–44	24–34
Contact method	49.0108	47.7008	49.2332
Fitting method	49.0097	47.7058	49.2290
Absolute value of difference	0.0011	0.0050	0.0042

The mean difference of the straight-line distance for the remaining seven pairs of center points in three directions, X, Y, and Z, was 0.0737 mm, 0.1683 mm, and −0.0030 mm, respectively.

Measurement values and differences of the straight-line distance of the remaining seven pairs of center points of the contact method and fitting method in three directions, X, Y, and Z (Tables 2–4).

**Table 2:** Measurement results of the straight-line distance at the X-axis (unit: mm).

	12–42	13–43	15–45	21–31	22–32	23–33	25–35
Contact method	1.9849	1.9816	2.4152	1.7177	1.0714	1.8891	2.9467
Fitting method	1.9963	1.9722	2.3048	1.6211	1.1297	1.7878	2.6787
Difference	-0.0114	0.0094	0.1104	0.0966	-0.0583	0.1013	0.2680

**Table 3:** Measurement results of the straight-line distance at the Y-axis (unit: mm).

	12–42	13–43	15–45	21–31	22–32	23–33	25–35
Contact method	2.8968	2.3439	2.0848	3.2969	3.9479	3.293	2.8224
Fitting method	2.8717	2.2487	2.0505	3.0915	3.4059	3.1546	2.685
Difference	0.0251	0.0952	0.0343	0.2054	0.5420	0.1384	0.1374

**Table 4:** Measurement results of the straight-line distance at the Z-axis (unit: mm).

	12–42	13–43	15–45	21–31	22–32	23–33	25–35
Contact method	49.3165	49.3061	45.3691	49.0629	49.7755	48.8441	44.4765
Fitting method	49.3246	49.2766	45.5671	48.9960	49.5093	48.8524	44.6456
Difference	-0.0081	0.0295	-0.1980	0.0669	0.2662	-0.0083	-0.1691

The $\bar{X}\pm S$ values were 0.0737 ± 0.107 mm, 0.1683 ± 0.176 mm, and −0.0030 ± 0.155 mm, respectively (Table 5). The measurement values of the straight-line distance of the remaining 7 pairs of the contact method and fitting method center points in three directions, X, Y, and Z, were respectively imported into SPSS 13.0. The paired *t*-test was used to compare the data of these two methods. Statistical significance was not observed for the X- and Z-axes, which represented the horizontal left/right and vertical directions, respectively. Statistical significance was observed for the Y-axis, which represented the horizontal anterior/posterior direction.

**Table 5:** Results of the paired-samples *t*-tests.

	X-axis distance	Y-axis distance	Z-axis distance
$\bar{X}\pm S$	0.0737 ± 0.107	0.1683 ± 0.176	−0.0030 ± 0.155
*p* value (two-tail)	0.119	0.045	0.961

## Discussion

The basic principle of the iterative closest point (ICP) algorithm is to roughly assign an initial alignment condition in two 3D models. ICP iteratively searches for the rigid transformation between the two, and uses minimum alignment error to achieve registration of the spatial geometric relationship between them. The ICP registration technique iteratively searches the nearest corresponding points, creates a transformation matrix, revises the transformation of one point until it reaches a convergence condition, and then the iteration stops [4]. Currently, commonly used 3D scanning systems in dental models are those of 3Shape, SmartOptics, and Dental Wings, among others [5, 6]. Their 3D scanning reconstruction of models with jaw relation is achieved based on scanning of the complete common area of the labial/buccal surface of the models with jaw relation and ICP registration of massive points.

Reference point system alignment (RPS) is referred to as being based on at least 3 pairs of reference points, by moving one or more objects in order to share location coordinates. In RPS, two objects are aligned by declaring a set of corresponding points on each and constraining each pair of points in a single direction. The direction of constraint for each pair can be the X-, Y-, or Z-axis, or a user-specified direction, and each pair can have a different direction. Reference points were respectively derived from the contact method and fitting method, each with existing measurement errors. Results indicated that the mean absolute value of the difference of straight-line distance between the three aligned pairs of reference points was 0.0034 mm, suggesting a high alignment accuracy. In this study, the weights of the three pairs of reference points used in the alignment were set to the same value. Therefore, in the alignment process, the effect of the three pairs of reference points at registration on jaw relation reconstruction was averaged. Meanwhile, in order to reduce alignment error, three pairs of corresponding positions, 11–41, 14–44, and 24–34, in the anterior region and bilateral molar region were selected as reference points for alignment, and the alignment errors were averaged to the whole dental arch.

Due to the rapid advances in the 3D construction [7, 8], the applications of 3D models have spread widely, including 3D graphics, CAD. The fundamental requirement of digitalization of computer denture is the accurate 3D reconstruction of the vertical distance and centric relation of digital edentulous jaw models. In this study, we use the contact measurement and the reference point system which was applied in different areas of the three dimensional measurement and recognition technology, such as mobile visual location recognition [9–11] and the evaluation of the accuracy of 3D location for big size object. The advance of computing techniques, including the mobile visual search [9–15] and augmented reality [16] will facilitate further research in CAD systems of complete denture.

Currently, the hardware and software systems for CAD and manufacturing of complete dentures are at the research and development stage ([17–20], S1 Video). Accurate 3D reconstruction of jaw relation in an edentulous jaw model is the upstream key link in CAD systems of complete denture. Being different from the dentition gypsum model, the characteristic geometric morphological area with obvious curvature changes in the edentulous alveolar ridge surface is lacking, which inevitably affects the accuracy of the ICP registration algorithm of the 3D reconstruction of jaw relation. Meanwhile, when employing the ICP algorithm of the labial/buccal common surface for 3D reconstruction of edentulous jaw model with jaw relation, a total of five objects (upper jaw model, upper jaw dentures, lower jaw model, lower jaw denture, and the labial/buccal surface of the models with jaw relation) need to be scanned respectively, making the process complicated. By using the Faro multiple degrees-of-freedom mechanical contact measurement system, the highly accurate spatial coordinates of any point within a certain area can be rapidly obtained. The 3.00-mm zirconium oxide probe of the measurement arm can determine the center point of the positioning cylinder distal surface, and the measurement error can be controlled within 10–40 μm. The results of this study showed that by applying three pairs of center points in the upper and lower jaw measured by this system, and only scanning upper and lower jaw models, the 3D reconstruction of edentulous jaw models with jaw relation can be achieved very efficiently, simplifying the reconstruction procedure.

The permitted error range of the jaw relation of complete denture system is usually 0.5–1.0 mm. In the current study, we applied multiple marker point contact measurement to achieve 3D reconstruction of edentulous jaw model with jaw relation. After reconstruction, the mean differences of the paired measurement points of the upper and lower jaw models in horizontal left/right direction (X-axis), horizontal anterior/posterior direction (Y-axis), and the vertical distance (Z-axis), were all within 0.2 mm, among which the error of vertical distance was less than 0.05 mm, which met the accuracy requirement for CAD of complete dentures. The standard deviations were all larger than 50 μm, suggesting that the accuracy of jaw relation reconstruction varied among segments. We determined that the reason for this was model rotation in 3D reconstruction. Rotation angle analysis should be emphasized in future studies, which can further enrich the evaluation index of 3D quantitation of edentulous jaw model with jaw relation.

The Faro multiple degrees-of-freedom contact measurement system used in this study is relatively rarely used in the field of dentistry, and the equipment is more expensive than other alternatives. Further studies should aim to define a less expensive method with fewer steps for 3D reconstruction of edentulous jaw digital model in centric relation.

## Supporting Information

S1 Video Automation of the teeth arrangement. One of the fundamental requirements of the digital complete denture is the automation of the teeth arrangement which is being researched at present.
(MP4)
